# Detection-Based Object Tracking Applied to Remote Ship Inspection

**DOI:** 10.3390/s21030761

**Published:** 2021-01-23

**Authors:** Jing Xie, Erik Stensrud, Torbjørn Skramstad

**Affiliations:** 1 Group Technology and Research, DNV GL, Veritasveien 1, 1363 Høvik, Norway; erik.stensrud@dnvgl.com; 2 Department of Computer Science, Norwegian University of Science and Technology, NO-7491 Trondheim, Norway; torbjorn.skramstad@ntnu.no

**Keywords:** object detection, object tracking, deep neural network, remote ship inspection

## Abstract

We propose a detection-based tracking system for automatically processing maritime ship inspection videos and predicting suspicious areas where cracks may exist. This system consists of two stages. Stage one uses a state-of-the-art object detection model, i.e., RetinaNet, which is customized with certain modifications and the optimal anchor setting for detecting cracks in the ship inspection images/videos. Stage two is an enhanced tracking system including two key components. The first component is a state-of-the-art tracker, namely, Channel and Spatial Reliability Tracker (CSRT), with improvements to handle model drift in a simple manner. The second component is a tailored data association algorithm which creates tracking trajectories for the cracks being tracked. This algorithm is based on not only the intersection over union (IoU) of the detections and tracking updates but also their respective areas when associating detections to the existing trackers. Consequently, the tracking results compensate for the detection jitters which could lead to both tracking jitter and creation of redundant trackers. Our study shows that the proposed detection-based tracking system has achieved a reasonable performance on automatically analyzing ship inspection videos. It has proven the feasibility of applying deep neural network based computer vision technologies to automating remote ship inspection. The proposed system is being matured and will be integrated into a digital infrastructure which will facilitate the whole ship inspection process.

## 1. Introduction

The rapid development and effectiveness of convolutional neural networks (CNN) have greatly contributed to enormously emerging computer vision applications and tools. As the performance and maturity level of computer vision technologies are continuously improving, we can utilize them to enhance or assist human tasks under certain circumstances in order to reduce safety risks, increase work efficiency, and cut costs.

One growing trend of applying computer vision technologies is remote visual inspection (RVI) [1,2], where a human inspector inspects a video instead of being physically present on the site. This is very beneficial when the assets to be inspected are difficult to access or in dangerous environments. Typical applications for RVI include aircraft and spacecraft engines, oil and gas pipelines, nuclear power stations, etc.

In recent years, applying RVI to ship inspection is of great interest to the maritime sector, in particular for inspection inside cargo and ballast water tanks. Regular ship inspections are required by the International Maritime Organization (IMO) with a specific purpose: “All ships must be surveyed and verified so that relevant certificates can be issued to establish that the ships are designed, constructed, maintained and managed in compliance with the requirements of IMO” [3]. The traditional way of conducting ship inspections is that human inspectors travel to the location of the ship and manually inspect all required areas. The whole process is costly, and human inspectors are often exposed to risky situations, such as shortage of oxygen in the tank, chemical toxicity, or other harsh environment. To reduce the inspection cost and increase personnel safety, leading ship classification societies (A ship classification society (https://en.wikipedia.org/wiki/Ship_classification_society) is a non-governmental organization that establishes and maintains technical standards for the construction and operation of ships and offshore structures.) such as DNV GL (DNV GL (www.dnvgl.com) is an international accredited registrar and classification society and headquartered in Høvik, Norway.), ABS (ABS (https://ww2.eagle.org/en.html) is an American maritime classification society.), and LIoyd’s Register (LIoyd’s Register Group Limited (https://www.lr.org/en/) is a technical and business services organization and a maritime classification society registered in England and Wales.) have been actively undertaking remote inspection services [4,5,6]. Remote inspection is becoming increasingly popular in times of the coronavirus crisis in 2020 [7]. However, the current practice of remote ship inspection still requires that the human inspectors visually inspect all of the streamed videos and manually identify the possible defects. The human inspectors have to concentrate on watching the several hours of inspection videos which often contain only a few minutes capturing the actual defects such as crack, corrosion, deformation and leakage. Such human-based visual inspection of videos is tedious, and the inspectors may therefore overlook some defects.

To further improve the remote ship inspection efficiency and increase inspection coverage as well, integrating computer vision technologies into a drone-based ship inspection process to automate the defect detection is promising [8]. One pragmatic scenario is that the inspection videos taken by the drone flying in the ship tanks are streamed to the cloud storage operated by the classification society. The videos, stored in the cloud, will be processed in a computer vision-based inspection system to automatically detect a variety of defects typically occurring in ships. The defects detected by the automated inspection system will be visualized in human understandable ways, such as using a bounding box to annotate the defects. Among those typical defects, cracks are most severe and have to be repaired before the ship can continue operating.

Another issue arising with the automated detection of defects in videos rather than in single images, is that many video frames will capture the same defect which will be detected many times as well. To save video inspection time, it is therefore also important to recognize the same defect across video frames, in order to reduce the number of frames that need to be inspected. An object tracking approach therefore adds the value to the automated inspection system by further reducing the number of video frames to be inspected.

In this paper, we present a detection-based object tracking system which automates the process of detecting cracks in the ship inspection videos and assists human inspectors to focus their attention on a subset of the video frames for analysis and decision-making. This system consists of two main modules concatenated. The first one is a CNN-based object detection model which detects cracks captured in the ship inspection images/videos. The second one is an object tracking module which tracks the crack detected by the detection model over the consecutive frames and maintains a unique index for the same crack.

The proposed detection-based crack tracking system has several distinguished features, i.e., our main contributions, listed below.

- The object detection module is customized to detect cracks in ship inspection images or videos. It is based on the RetinaNet model [9] with three major customizations presented in Section 3.3 and trained on our ship inspection image dataset (This ship inspection image dataset cannot be publicly accessible due to confidentiality).
- The object tracking module is based on SORT (simple online real-time tracking) [10] whose original tracker, i.e., Kalman Filter, is replaced with the Channel and Spatial Reliability Tracker (CSRT) [11] in this paper.
- Each individual crack appearing in a ship inspection video is assigned a unique tracking index which can be used to denote the corresponding crack in the ship inspection reports. The index assignment and associating detection hypotheses into tracking trajectories are achieved through modifying the data association function used in SORT. These modifications are added to solve the detection jitter issue caused by the object detection module.

The subsequent part of this paper is organized as follows. The related work is briefly summarized in Section 2. In Section 3, the proposed crack detection system is described in detail. The experiment results are presented in Section 4. The discussion and conclusion are presented in Section 5 and Section 6, respectively.

## 2. Related Work

There have been attempts to explore image processing techniques and deep learning approaches to automate the process of detecting cracks in ship structures. A percolation-based crack detector is proposed to identify crack pixels in an image in accordance with a set of rules [12]. This crack detector requires tuning several parameters based on its defined rules. Thus, the generalizability of this crack detector is unclear.

A CNN-based method is proposed to detect hull-stiffened plate cracks [13]. The proposed CNN model was trained on synthetic data which emulated only limited crack locations and lengths. It is not applicable to detection of cracks occurred in the ships under operation.

In this paper, we focus on investigating the feasibility of the CNN-based detection model which is trained on the real crack images captured by our ship inspectors. In addition, the object detection model does not require tuning parameters when applying it to unseen images.

### 2.1. Object Detection

Object detection is one of the common object recognition tasks. The main objective of object detection is to locate the presence of specific objects with a bounding box and classify the located objects in an image [14].

CNN-based object detection has proven to be an effective solution for numerous computer vision applications. The most widely deployed object detection application is facial recognition [15,16,17]. Other well-known applications of object detection include video surveillance, image retrieval, and the self-driving system of autonomous vehicles [18,19]. CNN models have been developed surprisingly complex in order to achieve human level performance, i.e., the number of stacked layers increases. For instance, the ResNets (short for Residual Networks) family, which is commonly used as the backbone network by many state-of-the-art object recognition models, has three network architecture realizations including ResNet 50-layer, ResNet 101-layer, and ResNet 152-layer [20]. Even the simplest ResNet architecture, ResNet 50-layer, contains around 25 million trainable parameters [21]. Such complex model architectures have to rely on enormous training data to globally optimize the huge parametric space of the model in order to achieve acceptable performance levels.

The real applications often lack sufficiently labeled training data to effectively train those complex CNN models. Thank to the publicly accessible image datasets, it has become a common practice in computer vision community that the novel but complex CNN models are first (pre-)trained on some large-scale public datasets which have been collected extensively and are suitable for more general data-driven tasks. For a specific real application, the user (or data scientist) can use the pretrained CNN model as starting point and train the same model on a customized dataset which is generally a small-scale and specific to the real application. Such a technique is called transfer learning, which is the improvement of learning in a new task through the transfer of knowledge from a related task that has already been learned [22].

### 2.2. Object Tracking in Videos

Object tracking in videos is a classical computer vision problem. It requires solving several sub-tasks including (1) detecting the object in a scene, (2) recognizing the same object in every frame of the video, and (3) distinguishing a specific object from other objects, both static and dynamic. The main technical challenges related to object tracking are data association, similarity measurement, correlation, matching/retrieval, reasoning with “strong” priors, and detection with very similar examples [23].

Object tracking algorithms can be categorized as either detection-based or detection-free. Detection-based tracking algorithms rely on a pretrained object detector to generate detection hypothesis which are used to form tracking trajectories. Detection-free tracking algorithms require manual initialization of a target object in the first frame where it appears. Detection-based tracking algorithms are of high interest because they can automatically start to track newly detected objects and terminate tracking objects disappearing.

When a specific application does not have a big set of videos which can be used to train a deep learning based object tracking model, the tracking approaches based on conventional computer vision technologies such as motion model and visual appearance model are more pragmatic. For instance, SORT was initially designed for online multiple object tracking (MOT) applications [10] and ranked as the best *open source* multiple object tracker on the 2D MOT benchmark 2015. It mainly consists of three components as listed below.

- An estimation model which is the representation and the motion model used to propagate a target’s identity into the next frame. The estimation model used in the original paper is Kalman Filter.
- A data association function which is to assign detections to existing targets. The assignment is solved optimally using the Hungarian algorithm (The Hungarian method is a combinatorial optimization algorithm that solves the assignment problem in polynomial time and which anticipated later primal-dual methods.).
- Creation and detection of tracker identities function which is to create or delete unique identities when objects enter and leave the image.

If an initial position of an object is given (e.g., a detection of an object), there are many modern and popular trackers which could provide good enough tracking performance. Particularly, OpenCV 4.4.0 (Open Source Computer Vision: https://docs.opencv.org/master/d9/df8/group__tracking.html) already provides APIs for several popular trackers including Boosting [24], CSRT [11], GOTURN [25], KCF [26], MedianFlow [27], MIL [28], MOSSE [29], and TLD [30].

## 3. Methods

### 3.1. Data Acquisition and Preparation

We have acquired a huge dataset of ship inspection images captured by our ship inspectors located all over the world. The dataset contains around 1.5 million images, of which the time span can be traced back to the early 2000 s. However, those images are not categorized and were not captured for machine learning purpose. The initial challenge was to identify which images are suitable for training a CNN model and then annotate them in a consistent way. The process of identifying proper crack images and annotating them is not only tedious and time consuming but also requires certain domain knowledge. Particularly, we could not outsource or crowdsource this task due to data confidentiality. We have been continuously working on it since 2017 and annotated around 4000 crack images so far. The details of identifying and annotating images are out of the scope and not discussed in this paper.

### 3.2. A Two-Stage Tracking System Overview

**Figure 1:**
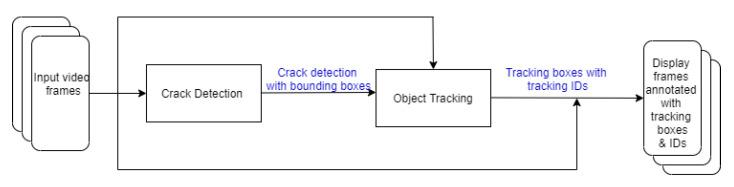
Detection-based crack tracking system.

We have acquired mainly static images and a few videos (either taken by human inspectors or drones). Therefore, it is not pragmatic to develop a CNN-based tracking-by-detection system which typically require a large video training dataset. Alternatively, we first use static images to train a CNN-based object detection model to detect cracks. Then, we propose a tracking model which uses the detection results as input to track cracks into consecutive frames. Such a two-stage system of detecting and tracking cracks is illustrated in Figure 1. Note that the *crack detection model* can be any object detection model. That makes the overall system flexible if we need to replace the existing crack detection model with another one which performs better.

### 3.3. Crack Detection Model—A Customized Convolutional Neural Network-Based Model

We initially investigated two state-of-the-art CNN-based object detection models, i.e., Faster R-CNN [31] and RetinaNet [9], which were trained using transfer learning to detect cracks occurring in the ship inspection images. The initial performance comparison showed that RetinaNet performed better on our customized data set which consists of around 1000 ship inspection images with annotated bounding boxes to locate cracks. Naturally, we chose RetinaNet as the benchmark detection model which could be replaced in future by the other one with better performance. Consequently, we continue exploring various techniques to further improve the performance of RetinaNet, including adaptive anchor box optimization and image augmentation.

The whole process of customizing the original RetinaNet to fulfil our needs includes three main steps as described below.

1. The preparation and preprocessing step uses image augmentation techniques to increase the training dataset and the anchor optimization method to find the optimal anchor setting for our crack images. More details are presented in Section 3.3.1 and Section 3.3.2, respectively.
2. We adopt the RetinaNet Keras implementation available in [32] and train it on the augmented training dataset. The RetinaNet model architecture can be found in the original publication [9] and thus is not repeated here. However, we observed that the RetinaNet Keras implementation often generates redundant bounding boxes around the same object in the image. To handle this issue, we propose a method to merge the overlapped predictions as presented in Section 3.3.3. The training configuration and results are presented in Section 4.1.1 and Section 4.1.2, respectively.
3. The test step is to evaluate the performance of the object detection model and recognize its advantages and limitations. The test configuration and results are presented in Section 4.1.3.

#### 3.3.1. Image Augmentation

**Figure 2:**
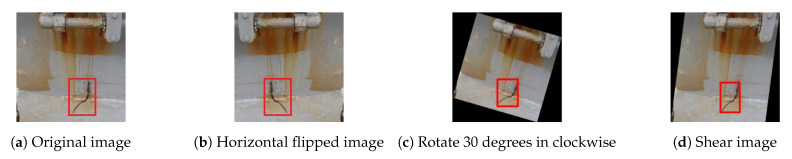
Image augmentation example: horizontal flip, rotation, and shear.

We have a small training dataset, i.e., initially around 1000 crack images. It is unlikely that using such small dataset to train RetinaNet which contains millions of trainable parameters could achieve the desired level of performance. Lack of sufficiently real data is a common issue for many deep neural network (DNN) applications. For instance, our application is dealing with the difficulty that crack occurrence in ships is a rare event. One effective way to combat insufficient training data and boost the performance of DNNs is data augmentation. Popular deep learning libraries like keras (Keras (https://keras.io/) is an open-source library that provides a Python interface for artificial neural networks. Keras acts as an interface for the TensorFlow library.), torchvision (TorchVision (https://pytorch.org/docs/stable/torchvision/index.html) is PyTorch’s own computer vision library which contains many important and useful datasets as well as models and transformations that are often used in the area of computer vision.) and specialized libraries on Github provide data augmentation for image classification training tasks. However, performing the image augmentation for an object detection task requires to update the bounding box accordingly. For example, Figure 2 shows that one image (Figure 2a) is horizontally flipped (Figure 2b) and the bounding box is transformed accordingly. Figure 2c shows the augmented image after rotating the image 30 degrees around the origin in clockwise direction. The resolution of the original image is maintained and the remaining area if any is filled by black color. A horizontal shearing shifts the upper part to the right and the lower part to the left as Figure 2d shows.

Data augmentation techniques can be categorized into three main types: geometric transformations, color space transformations, and photometric transformations [33]. We need to select the relevant ones for our dataset. After analyzing our crack image dataset, we identified that the orientation and size of the cracks are important factors. Moreover, we also would like to add some noise into the training data in order to force the CNN-based detection model to learn generalized features instead of overfitting on the small dataset. We selectively use seven augmentation techniques implemented in [34] to artificially expand our dataset, including horizontal flip, resize, HSV (Hue, saturation, value) conversion, rotation, scale, shear, and translation. The details of those augmentation techniques are introduced in [35].

#### 3.3.2. Anchor Optimization

Anchor boxes are fixed sized boxes that RetinaNet uses to predict the bounding box for an object. The anchor configuration is crucial for RetinaNet. The default anchor sizes (32, 64, 128, 256, and 512), aspect ratios (1:2, 1:1, and 2:1), and scales ($2^{0}$, $2^{\frac{1}{3}}$ and $2^{\frac{2}{3}}$) turn out to be ineffective for detecting objects of small size and large aspect ratios [36]. Cracks possess similar characteristics. Therefore, we employ the algorithm used in [36] to optimize aspect ratios and scales of anchors which best fit our crack image dataset. We aim to find the optimal anchor settings for scales and aspect ratios through maximizing the overlapping between the crack bounding-box and the best fitting anchor through analyzing the crack image dataset. The optimization results of anchor settings are summarized in Table 3, Section 4.1.1.

#### 3.3.3. Merge Overlapped Detections

Many object detection models possess a common problem that is generating redundant bounding boxes around the same object in the image. To solve this common issue, the non-maximum suppression (NMS) technique is often used to select one entity (e.g., bounding boxes) out of many overlapping entities. Although the RetinaNet implementation [32] used in this paper includes NMS as well, it sometimes does not remove all redundant detections. To whittle down the large number of redundant detection boxes to a few, we propose an algorithm to merge the overlapped detections. The algorithm is described in Appendix A.1.

### 3.4. Object Tracking

The training dataset is relatively small compared to the complexity of the object detection model. Thus, the performance of the crack detection is improved marginally by employing various fine-tuning techniques in addition to the customization techniques introduced in Section 3.3. Particularly, too many false positive detections are not acceptable if human inspectors have to verify each false detection manually. On the other hand, elimination of false negatives is even more critical. This is because negligence of actually existing cracks can lead to the wrong assessment of the ship condition and may cause risk of severe damage to the ship, whereas it is inevitable that CNN models are susceptible to prediction errors. Essentially we cannot eliminate the prediction errors of CNN models which are data-driven.

In an inspection video capturing cracks, one crack typically appears in multiple successive frames. Using the object detection model presented in Section 3.3 to detect cracks in the inspection videos typically ensures that the crack can be detected in some of the frames. However, we also have to accept that false detections may happen. To cope with the prediction errors of such CNN-based model we introduce object tracking into our crack detection system. The main purpose of using object tracking is to remove some false positive (FP) detections, fill the detection gap between true positives (TPs) (i.e., remove some false negatives (FNs)), and maintain a unique index for each individual crack.

#### 3.4.1. Tracking Process

**Figure 3:**
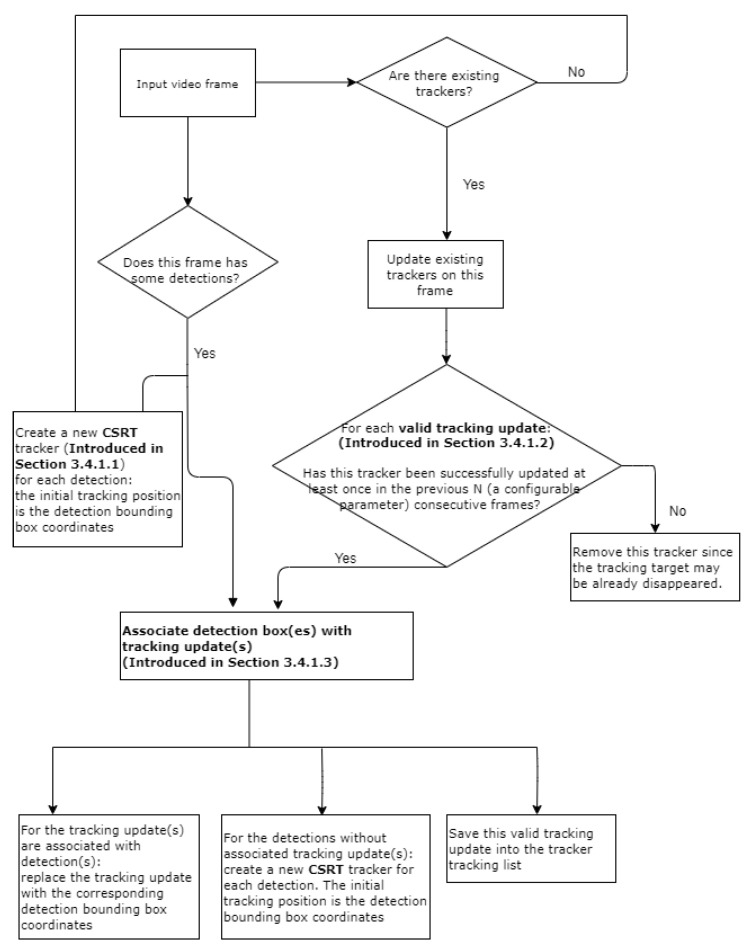
Process of object tracking.

The process of object tracking borrows the core concept of SORT [10] but are with several modifications as illustrated in Figure 3. Those modifications are explained in Section 3.4.1, respectively.

#### Using the CSRT Tracker to Track Potential Cracks

SORT uses Kalman filter to optimally solve the velocity components of the estimation model which decides how to propagate a target’s identity into the next frame. Such an estimation model is designed to build a motion model and is good at tracking moving objects, such as tracking pedestrians. However, SORT is not suitable for tracking cracks that are stationary and have arbitrary contours. We compared several trackers implemented in OpenCV and concluded that CSRT performs best on tracking cracks. CSRT has several strengths which are favorable for tracking cracks as listed below [37].

- Slower but more accurate than other popular trackers implemented in OpenCV (Refer those trackers listed in Section 2.2) [38].
- Robust to unpredictable motion.
- Trained on a single image patch.
- Can recover from failures when the object has not moved much.
- Can tolerate intermittent frame drops.

#### Postprocessing to Ensure Valid CSRT Tracking Updates

CSRT tracker has some limitations which hinder a robust crack tracking. Our observation of using CSRT implemented in OpenCV also reveals similar issues:

- it does not stop tracking when objects are no longer visible,
- it does not recover from multiple consecutive failed updates, and
- it latches onto surrounding regions when partially occluded resulting in drift.

To solve those limitations, we propose some postprocessing steps to ensure the valid tracking updates generated by the CSRT tracker as described in Appendix A.2.

#### Associate Detections with Existing Trackers

The most challenging part of multiple objects tracking is to associate detections with the existing trackers which is commonly known as data association problem in the relevant literatures. SORT creates an assignment cost matrix which computes the intersection-over-union (IoU) distance between each detection bounding box and all tracking updates from the existing trackers. The assignment is solved optimally using the Hungarian algorithm. Once a detection is assigned to an existing tracker, the current tracking update is replaced by the detection bounding box.

**Figure 4:**
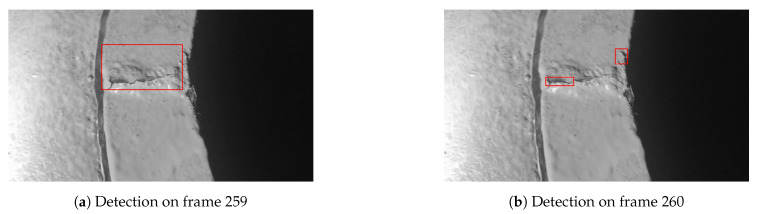
Scale of detection bounding boxes varies significantly on the consecutive frames.

However, we noticed that sometimes the scale of the detection boxes varies significantly in consecutive video frames. For instance, Figure 4 shows the detection results of two consecutive frames from a testing video file. The detection bounding box on frame 259 covers the major area of the crack, but the detection model located two separate areas on frame 260 and output two separate bounding boxes. For such case, using the detection bounding box to replace the tracking update not only causes severe tracking jitter but also requires creating a new tracker.

To solve this detection jitter issue, we propose a new algorithm to associate detections with the existing trackers as described in Appendix A.3. This algorithm consists of three steps to tackle the issue that the detections of the same crack may result in varying scales of detection boxes. More specifically, we first merge the small box(es) with the large box(es) if the small box(es) are within or majorly covered by the large box(es). This step guarantees that no bigger jitter happens during tracking process. Moreover, there is no need to create new trackers if more than one small detection boxes are within the same large tracking update. The second step is a data association function to address the issue that either the detection box or tracking update is relatively too large, and thus the IoU of these two boxes is smaller than the predefined IoU threshold. The core idea is to take into account both the intersection area and the area of the smallest box between the detection box and tracking update. Based on these two measurements we decide how to update the tracking box accordingly. The last step is to create new tracker per detection box which is not associated with any existing trackers yet.

## 4. Results

This section first presents the results of training and testing the customized object detection model introduced in Section 3.3. Due to the confidentiality of the ship inspection image dataset used for training such model, we can only provide some statistics of the training and test datasets with a limited number of image examples. Then, we present the results of applying the detection-based crack tracking system to the analysis of a ship inspection video.

### 4.1. Training and Testing the Customized Object Detection Model

#### 4.1.1. Training Process Description

Section 3.1 mentioned that categorizing and annotating the proper crack images are labor-intensive and require domain-specific knowledge. The training dataset has been iteratively increased as newly categorized crack images were annotated. The more training images are used for training the object detection model, the better performance results are achieved. As this paper focuses on the whole detection-based tracking system, we do not present all training history here but only the most recent training results.

The training, validation, and test datasets used in training and testing our object detection model are summarized in Table 1. The three datasets were from the same data collection which was randomly divided into three subsets corresponding to training, validation, and test sets. Therefore, we can assume that training, validation, and test sets come from the same distribution.

The definitions of these three datasets are given below [39].

- The training dataset is the sample of data used to fit the model.
- The validation dataset is a set of examples used to provide (unbiased) evaluation of a model fit on the training dataset while fine-tuning the hyperparameters of the model during the training process. The evaluation becomes more biased as skill on the validation dataset is incorporated into the model configuration.
- The test dataset is the sample of data used to provide an unbiased evaluation of a final model fit on the training dataset.

Using the validation dataset to evaluate the model occurs when every training epoch ends. Our object detection model essentially updates its hyperparameters based on the validation data evaluation results in an indirectly way.

Section 3.3.1 introduced that image augmentation is very useful for improving the model performance and robustness. In our training process, seven augmentation techniques are employed to transform one image into its varied versions: translation, shear, scale, rotation with nine various degrees, resize, horizontal flip, and HSV. The reason to rotate the image in nine various degrees is that we noticed the importance of the crack orientation in crack detection. However, the images in the training dataset do not provide sufficient crack samples with varied orientations. Thus, we algorithmically generate more training samples to mimic possible crack orientations. Note that image augmentation is applied to only the training dataset. After image augmentation, each training image has 15 augmented images additionally. The change of the training dataset is described in Table 2.

Training a deep CNN is computationally intensive and often executed using GPU(s). The training environment and configuration used in our work are described in Table 3. The RetinaNet model configuration were mostly set to default values such as the backbone network, initial weights, anchor sizes and strides, image maximum/minimum side, etc. However, the anchor ratios and scales were set to optimal values which were obtained from running the anchor optimization algorithm [36] on our crack image dataset (The dataset used for finding the optimal value of anchor ratios and scales was a subset of the training dataset described in Table 1. The reason is that the training dataset has been continuously updated until now but the optimal anchor settings have not been updated accordingly.). The number of training epochs was not very large as overfitting occurred in the early phase of training and continuously training longer will not improve the model performance anyway. The batch size is related to the computational resource limitation and can not be set to a too big value. The steps per epoch is calculated based on the number of training images and batch size.

#### 4.1.2. Training Results

**Figure 5:**
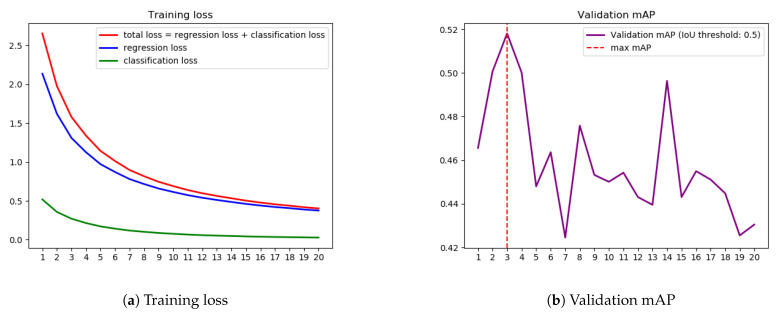
Training loss vs. Validation mean Average Precision (mAP).

We plotted the training loss and validation mean Average Precision (mAP) (Average precision is a popular metric in measuring the accuracy of object detectors. The detailed explanation can be found at: https://jonathan-hui.medium.com/map-mean-average-precision-for-object-detection-45c121a31173.) in Figure 5. The loss of object detection models consists of two parts: the regression loss for bounding box offset prediction and the classification loss for conditional class probabilities. Thus, the total loss is the sum of regression loss and classification loss. Figure 5a plotted three loss curves: regression loss, classification loss, and total loss. All three show that the training losses converged smoothly over time. However, the validation mAP plotted in Figure 5b fluctuates sharply and the best epoch result (the larger value means the better model performance) occurred at the 3rd epoch.

The training results reveal the model overfitting issue. The possible interpretations could be

- the training data points are not large enough compared to the model capacity (i.e., model is too complex and contains too many trainable parameters),
- wrong feature scaling/normalization was used, and
- no regularization technique was used.

#### 4.1.3. Testing Result

The trained weights of Epoch 3 was used to verify the model performance. The test dataset described in Table 1 contains 183 crack images and 248 ground-truth bounding boxes. Under the same GPU configuration as Table 3 describes, the total inference time of 183 images is 24.70 s. The test results are summarized in Table 4 where the IoU threshold used to assign ground-truth boxes to prediction boxes was varied between 0.1 and 0.6. When the IoU threshold was equal to 0.1, the model yielded the best test results such as the highest mAP and highest number of TPs. However, the crack detection model still predicted too many FPs, i.e., 7127 FPs when inferencing the test dataset. Although the desirable performance goal of the object detection model is to detect all existing cracks in the images, too many FPs are not acceptable since they need to be manually verified by the human inspectors.

**Figure 6:**
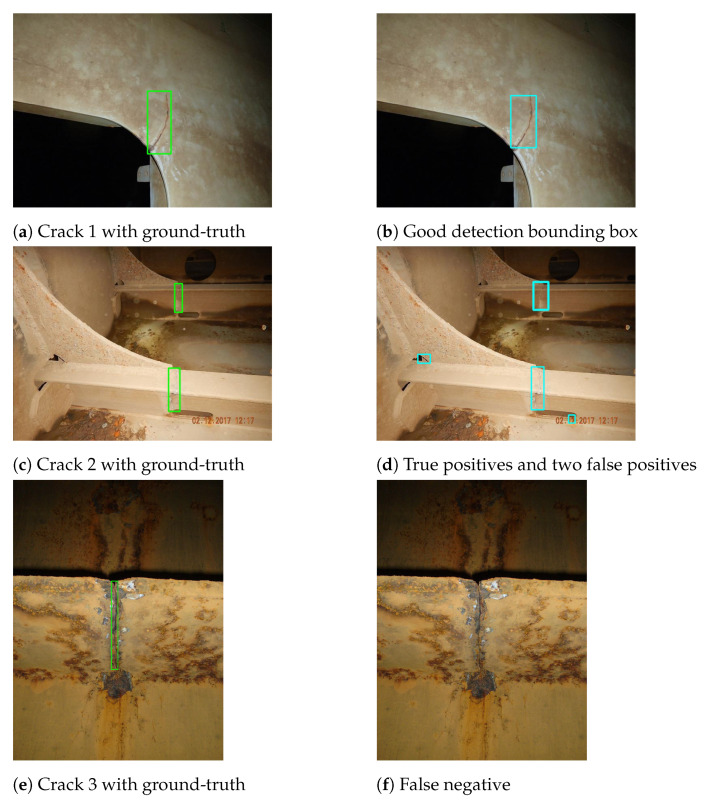
Examples of object detection test results.

We demonstrate several representative test images annotated with the predicted bounding boxes in Figure 6. The test data and training data are randomly selected from the same data collection as described in Section 4.1. Thus, we can assume that these two datasets follow the same distribution. The three examples included in Figure 6 demonstrate very diverse ship inspection situations and varied image qualities. Figure 6a represents a fairly good condition and the object detection model performed very well, i.e., Figure 6b shows a very good detection result where the predicted bounding box is tightly matched the ground-truth bounding box. However, when the background is very noisy such as Figure 6c represents, the object detection model predicted not only two TPs but also two FPs as shown in Figure 6d. The worst case is illustrated in Figure 6e where the crack appears in a heavily corroded condition. Then, the object detection model failed to detect the crack.

### 4.2. Object Tracking Test Results

The object detection model is used as an initial filter to locate suspicious areas where the cracks may exist. Then, the object tracking can further exclude some FPs and correlate TPs to remove intermittent FNs. Object tracking has to be applied to consecutive frame sequences with spatial and temporal correlations. We have collected a limited number of ship inspection videos taken either by our human inspectors manually or by flying drones. However, not all videos captured actual cracks as cracks rarely occur in ships. We selected one test video which captured an actual crack to examine the performance and effectiveness of our object tracking system as depicted in Figure 3. The test environment and system configuration are described in Table 5, where the parameters of object tracking are configurable and set to the empirical values.

Test Video A Results (use grayscale format to anonymize the test results presented in this section).

**Figure 7:**
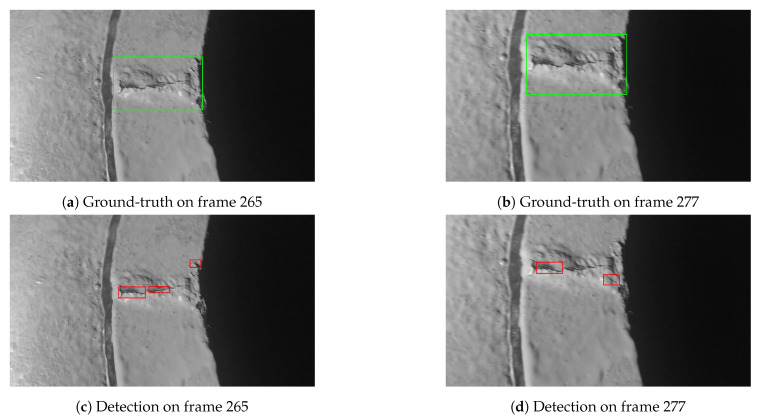
Object detection model occasionally predicted several small bounding boxes partly locating the crack.

Test video A was captured by a human inspector who was holding a digital camera and consciously kept a constant but close enough distance to the known crack while recording the video. The surface where the crack occurs looks fairly clean and in good condition. This video represents an ideal scenario where the object tracking system may perform well. The final tracking results are summarized in Table 6. Note that there is only one confirmed crack existing in the video. Thus, there is only one ground-truth bounding box per frame as illustrated in Figure 7a,b. The crack appears in multiple successive frames, i.e., between the 42th frame and 491th frame. However, the object detection model sometimes predicted several smaller bounding boxes which are around only part of the crack as indicated in Figure 7c,d. For such case, we counted those predicted bounding boxes as FPs if the IoU of the ground-truth box and each individual predicted bounding box is below the IoU threshold (i.e., 0.3) (Using the IoU of the ground-truth box and predicted bounding box decides whether a predicted bounding box is FP or TP is one of the commonly used criteria for assessing object detection models [40]. More specifically, if the IoU of the ground-truth box and predicted bounding box is below the IoU threshold, the prediction is considered as FP. Otherwise, the prediction is considered as TP.).

**Figure 8:**
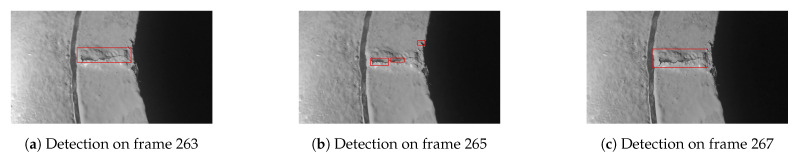
Object detection model is prone to detection bounding box jitters.

Figure 7c,d show that the object detection model indeed detected the crack. However, the predicted bounding boxes are too small compared to the predictions in the previous frames and successive frames as indicated in Figure 8. The predicted bounding boxes in Figure 8a,c also have small aspect ratio difference. In addition, we also observed that the object detection model did not predict any bounding box in some of the frames where the crack does appear. All those artifacts observed in the object detection results can be informally denoted as bounding box jitter [41].

The object tracking was applied to removal of not only false detections, but also smoothing detection bounding box jitters, i.e., the final tracking results should be robust and smooth. In addition, a unique index was assigned to each unique crack detected. The object tracking results of test video A are summarized in Table 6. The object tracking generated two tracking trajectories, Track ID 1 tracked the actual crack and Track ID 2 tracked a false crack. Compared to detection results, the tracking results achieved 27 more TPs and removed 17 FNs compared to the object detection results. It shows an increased robustness of the overall detection. The overall number of FPs is reduced to 24. However, we must highlight that the tracking approach also generated two new FPs associated with Track ID 2. The reason is that if the object detection model predicts FPs at the same location too many times (i.e., higher than some threshold defined in the object tracking system), the object tracking will consider those FPs as TPs. Then, the object tracking approach associated those FP detections into one tracking trajectory and connected the gaps between FPs to create a continuous trajectory. Thus, the root cause of reducing FPs is still in improving the performance of the object detection model.

**Figure 9:**
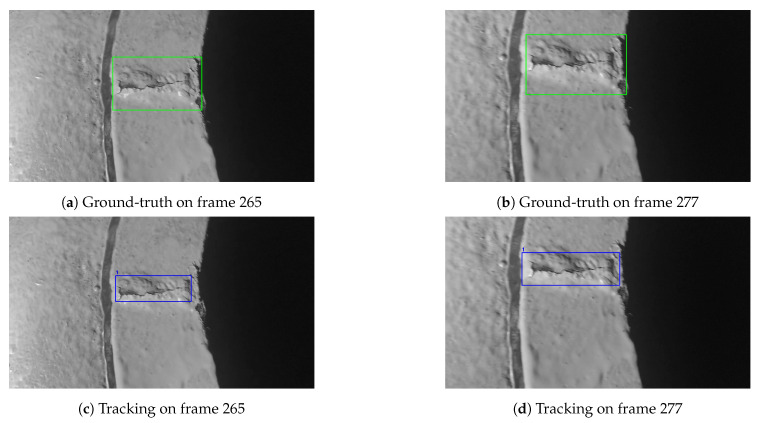
Tracking system removed detection jitter and predicted smooth tracking boxes.

It is worth highlighting that the object tracking system reduced some severe detection jitters and produced much smooth tracking results. For example, Figure 9 shows the same frames as Figure 7 does. However, tracking results in Figure 9 only generated one bounding box with an assigned ID 1 on both two frames. It means that the tracking system was tracking the same crack over frames.

**Figure 10:**
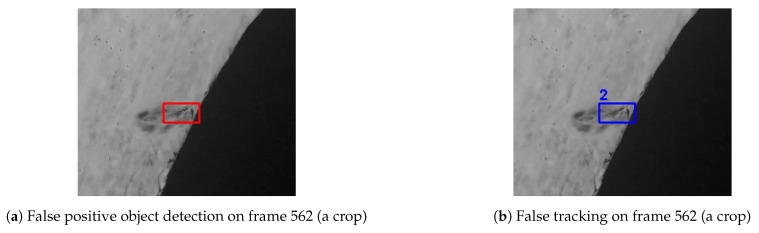
False detection and the corresponding tracking.

The FP detections are mainly concentrated on a specific location as shown in Figure 10. The object detection model predicted 19 FPs on this location in 34 consecutive frames (i.e., between frame 562 and frame 595), thus the object tracking system also considered this location containing a crack and associated multiple FPs with the same tracking ID 2. However, the human inspectors’ general feedback on such type of false detections is that it indicates some suspicious area and is worth investigating manually.

## 5. Discussions

### 5.1. Detection and Tracking Performance

There are some issues related to the performance. The first issue is that there are still too many FNs although the object tracking approach adds value and reduces the number of FNs. It is critical in our application to minimize the number of FNs as cracks compromise the structural integrity of the ship hull and therefore have to be spotted and repaired.

**Figure 11:**
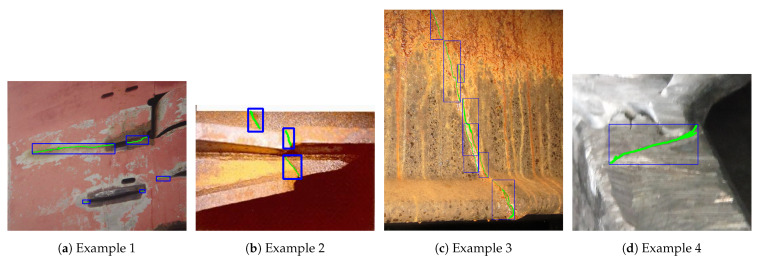
RetinaNet model training examples.

The second issue is that the customized RetinaNet model sometimes detects only a small part of the actual cracks and generates some severe detection jitters. One of the possible reasons is that the training images contain a diverse range of crack scales. In addition, the irregular shapes and orientations of cracks often lead to very loose ground-truth annotations when annotating training images. Figure 11 shows some training image example, where the actual cracks were first labeled using green color for pixel-wise representation (for training semantic segmentation model) and then are localized using the blue color bounding boxes (for training object detection model).

The third issue is related to the bounding box method. It is obvious that the actual cracks occupy only a small percentage of the bounding box areas. However, the RetinaNet has learned the whole bounding box as the potential “target object” which leads to a severe false positive learning. In Section 6, some further work is suggested to cope with these issues.

In addition, the detection speed of RetinaNet model varies significantly under different GPU configurations. For instance, when inferencing 183 static images with various shapes under the GPU configuration described in Table 3, it needed 24.70 s in total and led to the average inference time, 24.70 s/183 = 0.14 s. However, when inferencing the test video consisting of 595 frames with the same shape under the GPU configuration described in Table 5, the total inference time was 208.25 s and the average inference time was 0.35 s.

The tracking stage accuracy relies on the object detection model performance. The proposed tracking model can effectively fill the intermittent FNs in frames between two TP detections through data association. One important factor used to remove detection jitter is to consider both the IoU of the detection box and tracking box and their respective areas which is part of Algorithm A3. However, if the detection model predicts FPs at the same location more than certain numbers (some configurable parameter of the tracking model), the tracking model will lock this location and track it in the consecutive frames instead of removing those false detections. Thus, it requires both improving the detection model accuracy and employing other postprocessing techniques to identify and remove the false tracking.

### 5.2. Validity

The object detection model training results (refer Figure 5) shows that the model achieved the best training results at the completion of Epoch 3. One possible explanation is that using transfer learning with the pretrained backbone network has provided an initial set of the backbone network’s weights close to their optimal values. More specifically, the backbone network is responsible for extracting the low-level image features. Therefore, we argue that a crack is more likely an assembly of low-level features.

The object detection model were trained, validated, and tested on static images captured by human inspectors to document findings. Those images were not intended for training machine learning models. In contrast, the detection-based tracking system was tested on video footage captured by a dedicated human inspector who consciously emulated the drone flying movements, using a different camera and light source. It can be argued that the video footage used for testing the tracking system is partly out-of-distribution. Therefore, the overall performance results of the detection-based tracking system are conservative. One should expect improved detection and tracking performance in the future if the object detection model is re-trained using video footages which are captured by following a standardized process.

The training data of the object detection model also includes augmented images. We argue that the augmented images and the video footage have different distributions. Accordingly, the performance results of the detection-based system are conservative. We expect that the performance of the object detection model will be improved in the future by training it on more video footages and fewer augmented images.

## 6. Conclusions and Further Work

In this paper, a detection-based tracking system to detect and track cracks in ship inspection videos was proposed. The detection stage is a customized RetinaNet model with the optimal anchor setting and a postprocessing algorithm to remove the redundant predictions. The tracking stage consists of two main components: (1) one is the enhanced CSRT tracker which predicts the tracking updates in the consecutive frames by providing an initial tracking target, and (2) the other is a novel data association algorithm which associates detections with the existing trackers and maintain tracking indices for each unique tracking trajectory.

Most object detection models are prone to bounding box jitter [41]. Particularly, we observed three noticeable artifacts in our test videos. First, the aspect ratios of predicted bounding boxes may vary significantly in consecutive frames. Second, the model may predict several small bounding boxes, each of which is around only a part of the crack. Third, the model may not predict the crack at all in some frames in the frame sequence containing the crack. The detection bounding box jitter directly impacts the tracking performance. To avoid propagating detection jitters in the tracking stage, we developed a novel data association algorithm which considers not only the IoU of detection boxes and tracking updates, but also their respective areas when associating them with the same tracking trajectory. Our data association algorithm compensates for detection jitters and avoids initiating new trackers for those fragmented detections which essentially locate a small part of the actual cracks.

This work has proven the feasibility of applying CNN-based computer vision techniques to remote ship inspection. Our study showed that the state-of-the-art object detection and tracking models and relevant algorithms have to be customized or enhanced to extract patterns and features which can properly represent the cracks existing in our images and videos. The customized RetinaNet model has achieved a reasonable detection accuracy acknowledged by our ship inspectors. The results also demonstrate that the object tracking module adds value in reducing the number of FPs and FNs.

### Further Work

To mature this model and facilitate its usage in a production ready system, we need to further remove FP detections. There are several improvements to be considered.

First, we may improve the RetinaNet detection model’s performance and combat overfitting by adding more training data, fine-tuning model hyperparameters, or using regularization techniques such as dropout.

Second, semantic segmentation models can learn the features of cracks in pixel-level. In particular, horizontal bounding boxes have some limitations for training a crack detector. Cracks often appear at different scales and as irregular shapes (e.g., mostly as lines), and the pixels representing the crack constitute a small percentage of all the pixels within the bounding box. This results in the ground-truth annotations containing a large percentage of false positive pixels. Accordingly the object detection model trained on the dataset with such ground-truth annotations has learned some wrong features. We have been investigating U-Net model [42], which was originally developed for biomedical image segmentation purpose, in parallel with the RetinaNet model. Ensemble learning uses multiple learning models and combine multiple hypotheses generated by those constituent models to form a better one. RetinaNet model is relatively heavy and highly relies on the computing power. When applying this model to analysis of large volume of inspection videos, each of which may last a couple of hours, it will require substantially more processing time. Thus, it will be desirable to efficiently integrate both RetinaNet and U-Net models into the detection-based tracking system to improve the overall detection accuracy and robustness while not compromising the detection speed too much.

Third, we have been continuously identifying/annotating more training images/video footages using semisupervised learning to increase our training dataset size.

To further enable an automated inspection process, we are also developing a modular infrastructure, where data acquisition, data process, data storage, data visualization, and inspection reporting will be integrated. The proposed detection-based tracking system will be the main component of the data process module.

## Figures and Tables

**Table 1:** Summary of training, validation, and test datasets.

	Training Data	Validation Data	Test Data
Number of images annotated by 2019	1025 (91.5%)	45 (4.1%)	49 (4.4%)
Number of images annotated in 2020	1065 (80%)	133 (10%)	134 (10%)
Total number of images in the dataset	2090 (85.28%)	178 (7.26%)	183 (7.46%)
Number of ground truth bounding boxes in the dataset	3177	245	248

**Table 2:** Comparison of original and augmented training data.

	Original Dataset	Augmented Dataset
Number of images	2090	33,440
Number of ground truth bounding boxes in the dataset	3177	50,654

**Table 3:** Object detection model training environment and configuration.

Training model	RetinaNet Keras implementation
	Available on https://github.com/fizyr/keras-retinanet
RetinaNet model configuration	Backbone network: ResNet-50, Initial weights: Imagenet weights
	Anchor setting: Sizes-[32, 64, 128, 256, 512]
	Strides-[8, 16, 32, 64, 128]
	Ratios-[0.25, 0.556, 1.0, 1,797, 4.0]
	f Scales-[0.4, 0.521, 0.679, 0.908, 1.184]
	Image-max-side: 1333, Image-min-side: 800
Training configuration	Training epochs: 20, Steps per epoch: 25327, Batch-size: 2
	Label file: 1 class (i.e., Crack)
Deep learning framework	Tensorflow version: 1.15.2, Keras version: 2.3.1
GPU configuration	GeForce GTX 1080 Ti Graphic card, CUDA 9.2, cuDNN 7.6.4

**Table 4:** Customized RetinaNet Test results (using Epoch 3 trained weights, actual positives: 248). GPU configuration: same as Table 3. Total inference time of 183 images: 24.70 s.

IoU Threshold	0.1	0.2	0.3	0.4	0.5	0.6
Total detection	7351	7351	7351	7351	7351	7351
TPs	224	218	210	202	179	132
FPs	7127	7133	7141	7149	7172	7219
mAP	0.64	0.61	0.55	0.47	0.30	0.17
Precision	0.031	0.030	0.029	0.027	0.024	0.018
Recall	0.903	0.879	0.847	0.815	0.722	0.532

**Table 5:** Object tracking test environment and configuration.

Object Detection Model	RetinaNet (trained on crack images): trained weights of Epoch 3
Object Tracking Configuration (Empirical Values)	max_age=3
	Allow maximum 3 consecutive frames without being updated
	max_iou_threshold=0.8
	If the IoU of the detection box and tracking update is greater than this threshold, use the intersection area as the updated tracking box
	min_iou_threshold=0.3
	If the IoU of the detection box and tracking update is between min and max IoU thresholds, use the largest box as the updated tracking box
GPU Configuration	Quadro P620 Graphic card, CUDA 9.0, cuDNN 7.6.4

**Table 6:** Object tracking test video A results (one confirmed crack existing in the video).

Number of frames in the video	595
Inference time:	Total: 208.25 s, Average: 0.35 s
Number of ground-truth bounding boxes	450 (from frame 42 to frame 491)
	Frame range with detections: 45th–595th
	Number of actual detections: 459
Object detection results	Number of TPs: 412
(IoU threshold = 0.3)	Number of FPs: 47
	Number of FNs: 28
	Precision: 89.76%, Recall: 93.64%
	Frame range with tracking: 45th–585th
	Number of total tracking updates: 463
	Number of TPs: 439
Object tracking results	Number of FPs: 24
(IoU threshold = 0.3)	Number of FNs: 11
	Precision: 94.82%, Recall: 97.56%
	Number of tracking indices: 2
	Track ID: 1 (tracking actual crack from frame 45 to frame 483)
	Track ID: 2 (tracking false crack from frame 562 to frame 585)

## Data Availability

Data sharing not applicable

## Abbreviations

The following abbreviations are used in this manuscript:

CNN	Convolution neural network
CSRT	Channel and Spatial Reliability
DNN	Deep neural network
FN	False negative
FP	False positive
GPU	Graphics processing unit
HSV	Hue, saturation, value
IMO	International Maritime Organization
IoU	Intersection over Union
mAP	mean average precision
MOT	Multiple object tracking
NMS	Non-maximum suppression
ResNet	Residual Networks
RVI	Remote visual inspection
SORT	Simple online real-time tracking
TP	True positive

## Appendix A. Algorithms

### Appendix A.1. Merge Overlapped Detections

**Algorithm A1:** Merge overlapped detections.

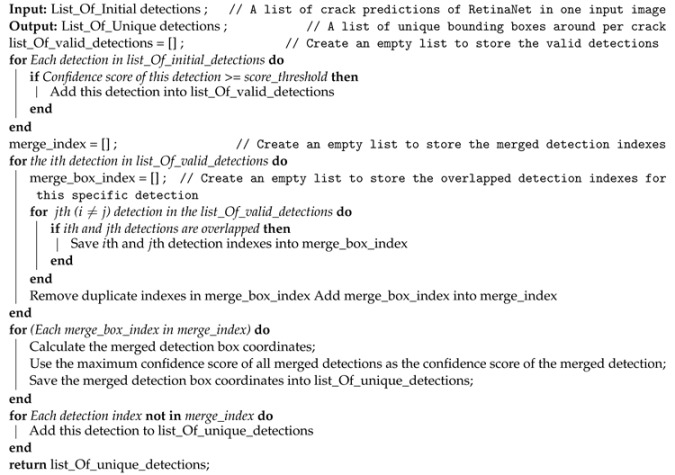

### Appendix A.2. Postprocessing of CSRT

**Algorithm A2:** Post-processing of CSRT to ensure valid tracking updates.

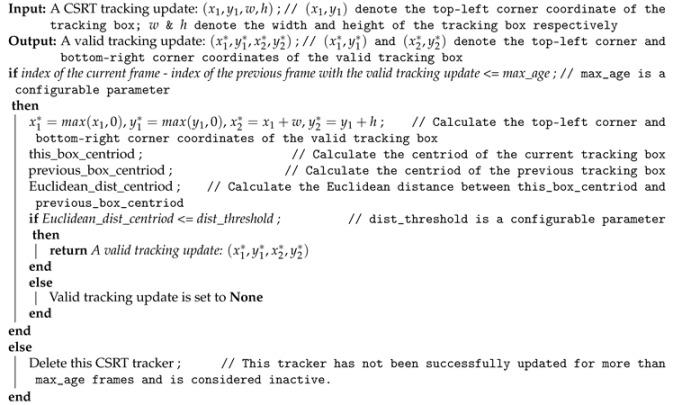

### Appendix A.3. Associate Crack Detections with Crack Trackers

Note that the IoU thresholds and *area_coefficient* defined in Algorithm A3 are empirical values and within the range of (0,1).

**Algorithm A3:** Associate crack detections with crack trackers.
